# Sustainable Electrospun Hybrid Nanofibers for Triboelectric Nanogenerators

**DOI:** 10.1002/smll.202410271

**Published:** 2025-01-29

**Authors:** Sweetly Thomas‐Kochakkadan, Marcos Duque, Gonzalo Murillo, Viraj P. Nirwan, Amir Fahmi

**Affiliations:** ^1^ Rhine‐Waal University of Applied Sciences Faculty of Technology and Bionics Marie‐Curie‐Straße 1 47533 Kleve Germany; ^2^ Instituto de Microelectrónica de Barcelona IMB‐CNM (CSIC) Til·lers s/n, Campus UAB Bellaterra 08193 Spain

**Keywords:** electrospinning, hybrid nanofibers, recycling, renewable energy harvesting, sustainability, triboelectric nanogenerators

## Abstract

Triboelectric nanogenerators (TENGs) have emerged as potential energy‐harvesting modules for miniaturized devices. TENG modules are derived often from components having low sustainability whereas the current environmental and economic circumstances demand a focus on sustainable, ecologically friendly approaches for the development of advanced hybrid materials. Herein, recycled polyethylene terephthalate (PET) along with commercially available nylon are electrospun into nanofibers for TENG devices. The obtained nanofibers are characterized using microscopy, spectroscopy, and thermal and mechanical analysis. Electrospinning of pristine and titanium dioxide nanoparticles (TiO_2_ NPs) blended polymer solutions resulted in uniform nanofibers without beads. The addition of TiO_2_ NPs improved the thermal properties and significantly improved the mechanical stability of the nanofibers. The performance of the fabricated TENG device has been improved by functionalizing the nanofibers with TiO_2_ NPs. Particularly, the combination of pristine PET and TiO_2_ NPs (5%) functionalized nylon nanofibers reached a peak power density of 23.44 mW m^−^^2^ with a surface charge density of 6.81 µC m^−^^2^, a max output voltage of 111 V and a max current of −1.61µA. This study opens a new avenue to utilize upcycled cost‐effective polymers processed using electrospinning as a powerful tool for the fabrication of the next generation of sustainable TENG devices.

## Graphical abstract

Fabrication of advanced hybrid materials from recycled polymer provides an environmentally friendly and economical platform for TENG. TENG is a sustainable approach to capture energy for a wide range of applications. Using electrospinning and recycled materials, a facile strategy is provided to fabricate hybrid nanofibers for efficient TENG devices. The TENG module can power health monitoring devices, charging capacitors, sensors, and batteries.

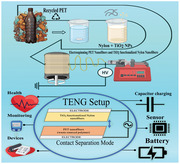

## 1 Introduction

Triboelectric nanogenerators are innovative energy harvesters that convert relative motion between two or more materials into electrical energy, utilizing the principles of contact electrification and electrostatic induction.^[^ ^1^ ^]^ This concept was first demonstrated in 2012 by Prof. Zhong Lin Wang and his team at the Georgia Institute of Technology.^[^ ^2^ ^]^ Since then, TENG‐powered devices have been proposed for applications such as human physiological monitoring, and sustainibility based energy generation.^[^ ^3^, ^4^, ^5^ ^]^

Briefly, when two triboelectric plates come into contact, friction causes the transfer of electric charges, ions, and materials between the plates. Upon separation, some charges remain on the plates, while others return to their original positions.^[^ ^1^ ^]^ This process generates triboelectric charges on both plates, creating a potential difference and enabling energy harnessing.

To maximize power output from a TENG, it is essential to use materials that can effectively take part in this charge‐transfer. Nylon 6,6, a low‐cost material, can serve as an electron donor when paired with a suitable triboelectric negative material, such as PET. PET is widely used in the plastic bottle industry and is both inexpensive and readily available. Utilizing waste PET bottles as the triboelectric negative material in TENGs offers a sustainable recycling solution. However, PET is less triboelectric negative than other materials like PTFE, polypropylene, or polyethylene, leading to suboptimal TENG performance.^[^ ^6^ ^]^ Strategies such as increasing the surface contact area or incorporating nanoscale materials like TiO_2_ NPs can be employed to enhance TENG efficiency. TiO_2_ NPs, known for their excellent dielectric properties, can improve the overall dielectric properties of the hybrid nanofiber matrix, thus enhancing TENG performance.^[^ ^7^, ^8^, ^9^ ^]^ Hybrid nanofibers functionalized with TiO_2_ NPs have been proven to be beneficial for a variety of applications including remediation of dyes, photocatalysis, and dye‐sensitized solar cells. For instance, the TiO_2_ NPs functionalization of polyamide (PA6) nanofibers provided them with improved dye absorbing (methylene blue) capacity.^[^ ^10^ ^]^ Similarly, TiO_2_ NPs based nanofibers are shown to be effective for the degradation of organic pollutants in water.^[^ ^11^ ^]^ The photocatalytic activity of TiO_2_ NPs has been combined with various metals and metal oxides for the degradation of dyes.^[^ ^12^ ^]^ Moreover, TiO_2_ NPs combined with carbon nanofibers are employed in Li‐S batteries as efficient interlayers offering good conductivity in the cathode region while maintaining the soluble polysulfides.^[^ ^13^ ^]^ The inclusion of TiO_2_ NPs in nanofibers matrix provides multifaceted effects, involving interfacial interactions and changes in surface charge characteristics. Primarily, the inclusion of TiO_2_ NPs enhances the mechanical properties via improved interfacial interactions between the polymer and nanoparticles.^[^ ^14^, ^15^ ^]^ A higher Youngs modulus and hardness associated with ceramic materials (TiO_2_ NPs) provide a reinforcing effect consequently, leading to load transfer and stress distribution more uniformly throughout the hybrid materials offering improved mechanical properties. Even more this effect is pronounced by the nanoscale dimension ensuring a high aspect ratio, which in turn leads to more interaction sites and a higher packing ratio when compared to the bulk material.^[^ ^16^ ^]^ Additionally, the TiO_2_ NPs demonstrate excellent dielectric properties, therefore allowing an improved polarization behavior in the functionalized materials, which is advantageous for applications in TENG where higher charge separation translates to improved TENG performance.^[^ ^17^, ^18^ ^]^ Moreover, TiO_2_ NPs are intrinsically n‐type semiconductors with a wide bandgap of ≈3.3ev, hence finding their applications in dye‐sensitized solar cells.^[^ ^19^ ^]^ Therefore, it plays a vital role in increasing the charge‐transfer efficiency of the functionalized nanofiber surfaces, making it suitable for TENGs.^[^ ^20^ ^]^

Several papers have emerged recently incorporating PET and nylon as triboelectric materials. In a 2024 study, Gulahmadove et al. investigated the enhancement of TENG by using nylon films coated with TiO_2_ NPs as a triboelectric material.^[^ ^21^ ^]^ The key findings of the study show that when the nylon film was sprayed 15 times with TiO_2_ NPs, the dielectric constant of the film increased by almost 50%.^[^ ^21^ ^]^ This dielectric increment resulted in the enhancement of the overall performance of the TENG, with output voltage and current increased by 36% and 37%, respectively.^[^ ^21^ ^]^ Another study conducted in 2022 by Bairagi et al. fabricated a TENG with silk and PET as triboelectric materials.^[^ ^22^ ^]^ The triboelectric properties of the materials were increased by coating electrospun nanofibers on the surface.^[^ ^22^ ^]^ Nylon 6,6 nanofibers were coated on the silk, and polyvinylidene fluoride nanofibers were coated on the PET films.^[^ ^22^ ^]^ The deposition of electrospun fibers on TENG surfaces improved the output voltage and current significantly, by ≈17and 16 times, respectively.^[^ ^22^ ^]^ Therefore, emphasizing the significance of possessing nanofibril structure on the surface for the development of efficient TENG. Further, the immobilization of TiO_2_ NPs has been shown to improve the functionality of nanofibers for multiple applications.^[^ ^6^, ^23^, ^24^ ^]^ Specifically, TENG devices have been shown to benefit from the inclusion of TiO_2_ NPs as functional agents.^[^ ^20^, ^23^, ^25^, ^26^ ^]^ Here, electrospinning is employed to fabricate nanofibers by blending various polymers along with TiO_2_ NPs to improve their collective properties. Electrospinning is an efficient method for producing extremely thin nanofibers from a polymeric solution, making it an effective technique for recycling waste PET bottles into PET nanofibers.^[^ ^27^ ^]^ These electrospun nanofibers possess a very high surface area, which enhances triboelectric charge separation compared to bulk materials. Briefly, the polymer is dissolved in a suitable solvent, and the resulting solution is subjected to a high electric potential to facilitate the fabrication of nanofibers. Additionally, electrospinning allows immobilization of NPs within the polymer matrix by simply suspending NPs in the polymer solution at appropriate concentrations.

In this report, the fabrication and testing of four TENG devices made from sustainably sourced PET and commercially available nylon 6,6 are presented. To achieve this, four combinations of triboelectric electrospun nanofibers were utilized: a pristine PET nanofiber mat, a hybrid PET nanofiber mat incorporating TiO_2_ NPs into the matrix, a pristine nylon 6,6 nanofiber mat, and a hybrid nylon 6,6 nanofiber mat containing TiO_2_ NPs within the nanofiber matrix. A vast number of characterization techniques were used to investigate the collective properties of the generated nanofibers and to evaluate the performance of the TENG devices.

## 2 Experimental Section

### 2.1 Materials

Trifluoroacetic acid (TFA) (≥99.5%), dichloromethane (DCM) (≥99.5%), ethanol (≥99.5%), nylon 6,6, and formic acid (90%) were supplied by Sigma–Aldrich, polyethylene terephthalate was obtained from a waste soft drink bottle. TiO_2_ NPs were prepared elsewhere. All chemicals were used as received.

### 2.2 Synthesis of Nanofibers

#### 2.2.1 Preparation of Pristine Nanofibers

To fabricate pristine PET (PP) nanofibers recycled bottles were shredded into small pieces, and then washed thoroughly with distilled water, followed by ethanol. The shredded PET was then dried at room‐temperature for 48 h to ensure the removal of any remaining water and ethanol. A 15% (w/v) PET solution was prepared by dissolving the shredded PET in a 3:2 ratio of TFA and DCM. This solution was then used to produce electrospun nanofibers.

For the fabrication of pristine nylon (PN) nanofibers, 15% (w/v) nylon 6,6 pellets were dissolved in 90% formic acid.^[^ ^28^ ^]^ The nanofibers were obtained by the electrospinning of the solution with conditions presented in **Table** **1**.

**Table 1:** Various electrospinning parameters are used to prepare the polymer nanofibers.

Sample Name	Voltage [kV]	Flow Rate [mL hr^−1^])	Temperature [°C]	Humidity [%]
Pristine PET (PP)	+ 16 / −4	0.3	30	35
Hybrid PET (PBHN)	+ 16 / −4	0.3	30	35
Pristine Nylon (PN)	+ 20 / −4	0.4	30	35
Hybrid Nylon (NBHN 2% / NBHN 5%)	+ 20 / −4	0.4	30	35

#### 2.2.2 Preparation of Hybrid Nanofibers

The shredded PET pieces and TiO_2_ NPs were mixed in TFA and DCM in a 3:2 ratio separately. Both the solutions were mixed after some time to get a final PET concentration of 15% (w/v) and a TiO_2_ NPs concentration of 2% (w/v) and the solution was electrospun to obtain PET‐based hybrid nanofibers (PBHN).

For the electrospinning to obtain nylon‐based hybrid nanofibers (NBHN 2%), the nylon 6,6 pellets and TiO_2_ NPs were mixed in 90% formic acid separately. The two solutions were mixed after 2 h to prepare 15% (w/v) nylon 6,6 and 2% (w/v) TiO_2_ NPs polymer solution. The specific parameters for electrospinning are given in Table 1.

To ascertain further the effect of TiO_2_ NPs’ addition to the nanofibers a composition that showed the most significant TENG performance was improved by increasing the ratio of polymer to NPs. Here, for the combination of PN/NBHN, TiO_2_ NPs in hybrid nylon were increased from 2% to 5% (w/v). The composition (NBHN 5%) was electrospun using the optimized parameters. The fibers were analyzed using ‐Attenuated total reflectance (ATR) Fourier transform infrared (FTIR), Thermogravimetric analysis (TGA) and Scanning electron microscopy (SEM). A detailed analysis of the nanofibers with increased NPs’ concentration can be found under Figures S1 and S2 (Supporting Information).

### 2.3 Characterization of TiO_2_ NPs and Electrospun Nanofibers

Young's modulus of the pristine and hybrid (polymer with TiO_2_ NPs) nanofibers were determined by calculating the ratio of yield stress to yield strain. These values were obtained using a Tinius Olsen UTM machine. The prepared samples had a width of 10 mm, a gauge length of 20 mm, and an average thickness of 150 µm.^[^ ^29^ ^]^ TGA was employed to study the thermal properties of both pristine and hybrid nanofibers. In the TGA (Perkin Elmer), the samples were weighed and then placed inside a ceramic crucible. The analysis involved heating the samples under nitrogen from 35 to 700 °C at a rate of 10 °C min^−1^.^[^ ^30^ ^]^ The rheological analysis of samples was performed by pressing the fibers in the form of a tablet with a diameter of 7 and ≈1 mm in height. Using parallel plate geometry in a Rheometric scientific rheometer the dynamic analysis of viscoelastic properties of samples was performed.

‐ATR‐FTIR (Perkin Elmer) spectroscopy was performed to identify interactions among constituents in the composition of the electrospun nanofibers, the scan was performed covering wavenumbers from 600 to 4000 cm^−^¹ at a resolution of 2 cm^−^¹.^[^ ^31^ ^]^ SEM (JSM‐IT 100 InTouchScope™) coupled with an energy‐dispersive X‐ray analyzer was used to visualize the morphology of the nanofibers and perform elemental analysis to confirm the presence of TiO_2_ NPs in the matrix of functionalized nanofibers. High resolution‐ transmission electron microscopy (HR‐TEM) was performed on TiO_2_ NPs suspended in dist. H_2_O using JEOL 2200 fs (HR‐TEM). ImageJ^®^ was used to perform the diameter analysis of nanoparticles and nanofibers from their respective micrographs. Finally, OriginLab was used to perform the statistical analysis along with preparation graphs and their presentation.

### 2.4 Triboelectric Tests

For the electromechanical characterization the setup was used as developed,^[^ ^32^ ^]^ different triboelectric materials (5 cm x 5 cm) are placed on top of two parallel test platforms (10 cm × 10 cm) made of polymethyl methacrylate (PMMA), with four metal guides and four springs to retract both surfaces after contact. To ensure the electrical connection 5 cm × 5 cm PMMA blocks covered by copper layers are used. Finally, these pieces are positioned between the parallel test platform, attached to each facing surface. In total, two electrodes and two dielectric materials are used (contact‐separation mode). The top electrode separated periodically after coming into contact with the bottom part to facilitate charge‐transfer. To carry out the electrical measurements, an ad‐hoc characterization setup was assembled, consisting of a stepper motor (Zaber LSQ075B‐T3‐MC03 and X‐MCB1‐KX13B), a dynamometer (Mark M5i and MR03‐20 sensor for a maximum force of 100 N), a sourcemeter (Keithley 2470), and a LabVIEW program that controls the entire electrical characterization setup. The measurements were conducted with a tapping frequency of 0.5 Hz and with a maximum contact force of 10–12 N.

The surface charge density of each TENG was calculated using the formula *Q* = ∫*I dt*.^[^ ^22^, ^30^ ^]^ Where *I* is the instantaneous current, and *dt* is the time differential. By dividing the surface charges by the surface area of the TENG will get the surface charge density. Moreover, the peak power output of the TENG is calculated by the equation *P_Peak_ = V_Peak_^2^ R^−1^*. Where *V_Peak_* is the peak voltage of the TENG and *R* is the impedance load.^[^ ^22^, ^30^ ^]^ On the other hand, the average power output of the TENG is calculated by the equation *P_avg_ = 1/Δt∫V^2^/R dt*. Where *V is the* instantaneous voltage generated by each TENG, *Δt* is the time interval and *R* is the load impedance.

## 3 Results and Discussion

**Figure 1:**
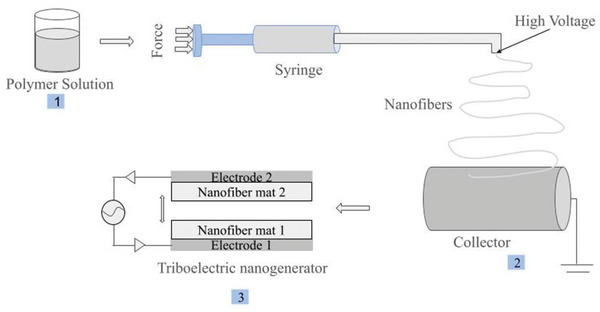
A schematic of all the processes involved in the fabrication of TENG. 1) In the first step the polymer is dissolved in a suitable solvent to prepare the solution. 2) The solution is transferred to a syringe. Where high voltage is applied at the tip of the needle, nanofibers start to emerge from there and are deposited at a rotating collector. 3) The nanofiber mat from the collector is removed and an electrode material is attached to the one end to create a triboelectric layer. The current generated when two triboelectric layers go through contact and separation cycles were measured across electrodes.

Electrospinning is a powerful technique for generating nanofibers.^[^ ^27^, ^33^ ^]^ It involves the application of a high voltage to draw a polymer solution or melt, into fine fibers. A schematic has been presented in **Figure** **1** to highlight the fabrication of the TENG device using electrospinning. The general high molecular weight polymers used for manufacturing PET bottles eased the fabrication of nanofibers. Therefore, once the parameters for electrospinning and solvent ratios for the dissolution of PET were optimized, the fabrication of the nanofibers was smooth. The blending of the TiO_2_ NPs with the electrospinning solution resulted in nanofibers with a yellowish color. Similarly, nylon has been used widely for the fabrication of nanofibers, using the parameters from the literature simply pristine and hybrid nylon nanofibers were obtained.^[^ ^28^ ^]^ The nanofibers were dried to remove the residual solvents at 30 °C in the oven for 2 days. The dried fibers were used for further analysis and characterization.

### 3.1 Microscopy Analysis

**Figure 2:**
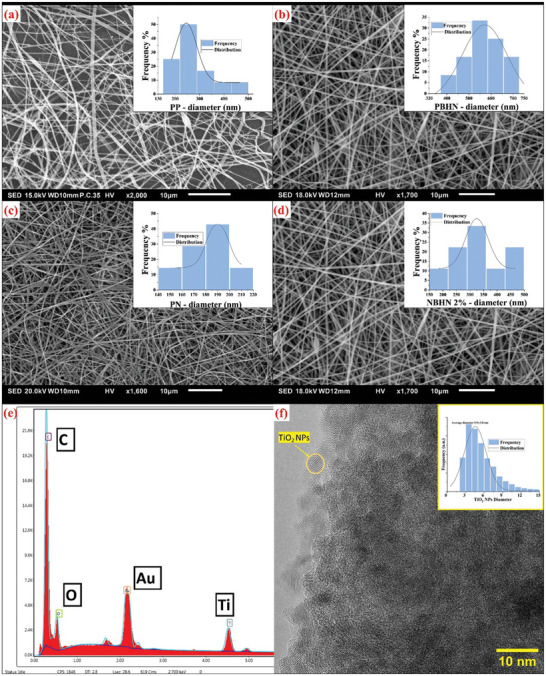
SEM micrographs of the nanofibers and the statistical analysis of the nanofiber diameter for PP nanofiber with an average diameter of 274 ± 92 nm a). When TiO_2_ NPs were added the average diameter increased to 565 ± 99 nm b), PN nanofibers had an average diameter of 180 ± 21 nm c), and the NBHN 2% nanofibers had an average diameter of 334 ± 88 nm d). EDX analysis of functionalized nanofibers is used to confirm the presence of TiO_2_ NPs. The signal for Au is due to the sputtering performed on the fibers for SEM preparation e). Diameter analysis of TiO_2_ NPs from micrograph obtained after HR‐TEM analysis revealed an average diameter of 5.9± 3.8 nm f).

Microscopy measurements of pristine nanofibers, hybrid nanofibers, their EDX analysis, and HR‐TEM analysis of TiO_2_ NPs are presented in **Figure** **2**. The SEM analysis of PP, PN, PBHN, and NBHN 2% mats was conducted to reveal the morphology of the fibers. When the concentration of PET solution was less than 15% (w/v), the unidirectional structures formed were mostly composed of beads with very little or no fibers.^[^ ^34^ ^]^ The lower concentration threshold for the formation of some fibers was 10% however, with many beads similar to the results published by Veleirinho et al.^[^ ^34^ ^]^ At 15% PET concentration, in contrast to the study conducted by Veleirinho et al., desired PP nanofibers with an average diameter of 274 ± 92 nm were obtained as shown in Figure 2a. This contrasting outcome could be the result of the variation in the molecular weight used in both studies. The addition of 2% (w/v) TiO₂ NPs increased the fiber diameter. The PBHN fibers showed an average diameter of 565 ± 99 nm.

Similar to that of PP nanofibers, when the concentration of the nylon solution was less than 15% non‐uniform nanofibers with beads were produced from the electrospinning process.^[^ ^28^ ^]^ At 15%, smooth fibers without beads were obtained as shown in Figure 2c.^[^ ^28^ ^]^ The average diameter of PN nanofibers is 180 ± 21 nm, while the NBHN 2% nanofibers have an average diameter of 334 ± 88 nm. In the case of nylon nanofibers, both the pristine and hybrid samples exhibited a lower standard deviation. Here, due to the lower magnification used for the performance of SEM analysis, the presence of TiO_2_ could not be determined. Expectedly, the size of the nanoparticles lies far lower and they were not resolved with SEM on the measured nanofiber samples. Therefore, elemental analysis of the functionalized NBHN 2% nanofibers was performed using the complementary EDX attached to the SEM. The elemental analysis confirmed the presence of titanium in the functionalized nanofibers along with the usual elements such as C, and O forming the backbone of the polymer fibers and Au used for sputtering the fibers before observing them under the microscope. Additionally, TiO_2_ NPs were analyzed using HR‐TEM, which helped determine their size distribution. By analyzing the obtained micrograph using the ImageJ software, the average diameter of the NPs was measured to be 5.9 ± 3.8 nm. The nanoparticles showed a tendency to be clustered pretty quickly in the absence of agitation such as using ultrasonication or simply stirring. This might be attributed to very high surface energy which prefers agglomeration of nanoparticles. Therefore, to minimize this effect during the preparation of the electrospinning solution, ultrasonication was performed before and after the addition of polymer to the nanoparticles, which led to stable solutions primarily, due to ultrasonication and high viscosity of the electrospinning solutions.

The hybrid nylon nanofibers electrospun with 5% w/v TiO_2_ NPs ratio demonstrated an average diameter of 382 ± 98 nm. As seen in Figure S1 (Supporting Information), compared to the morphology of the NBHN 2% sample, there was no discernable difference, and increasing the amount of TiO_2_ NPs had little effect on the overall shape  as observed under SEM.

### 3.2 Mechanical Properties

In the contact‐separation mode of TENG, the triboelectric materials are subjected to compressive stresses when the materials come into contact. During retraction, the same materials experience tensile stresses. To obtain a continuous power output from a TENG, the device must operate in the range of 5–10 Hz, which requires 300 to 600 contact separations per minute.^[^ ^35^ ^]^ Under these extreme working conditions, the triboelectric materials must have sufficient strength to prevent permanent deformations. Hence, measuring the mechanical properties and studying rheological behavior are important characterization techniques to investigate the collective properties of TENG devices.

The Young's modulus of each type of nanofiber mat was calculated using the data obtained from the analysis (S3). The PP nanofiber mat exhibits Young's modulus of 2.10 MPa, while the PBHN mat, with 2% (w/v) TiO₂ NPs, shows Young's modulus of 4.06 MPa. Similarly, the PN nanofiber mat has a Young's modulus of 3.9 MPa, which increases to 8.73 MPa in the NBHN 2%. The incorporation of TiO₂ NPs into the nanofiber matrix significantly enhanced the mechanical properties of the material. The addition of just 2% (w/v) TiO₂ NPs nearly doubled the Young's modulus of the nanofibers.^[^ ^36^ ^]^

**Figure 3:**
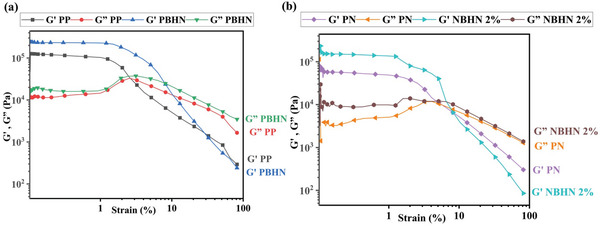
Results of strain sweep analysis of a) PP, PBHN, and b) PN, NBHN 2% nanofibers. Overall, the increased strain caused a crossover point of *G*′, *G*″ for all samples. The inclusion of TiO_2_ NPs shows an increase in the moduli of *G*′ and *G*″.

The viscoelastic properties of the nanofibers were measured using parallel plate geometry to identify their behavior under dynamic stresses.^[^ ^37^ ^]^ The results of the strain sweep of PP, PBHN, PN, and NBHN 2% samples are shown in **Figure** **3**a,b respectively. The tests were conducted at a temperature of 230 °C and a frequency of 2 Hz. As expected there is a large gap between the initial storage modulus (*G*′) and loss modulus (*G″*) in all the samples, and the elastic behavior of the polymers is dominant.^[^ ^37^ ^]^ As the strain is increased to 1%, *G*′ decreases rapidly followed by *G*″, indicating a transition toward the viscous dominant region with crossover being observed after the strain has increased beyond 10%.^[^ ^38^ ^]^ A similar, pattern was observed in *G*′ and *G*″ of the fibers in the presence of TiO_2_ NPs in the matrix. An increase in modulus can be observed in both PBHN and NBHN 2% as compared to PP and PN, respectively. The increment of *G*′ and *G*″ values in the presence of NPs is well‐documented in the literature, presumably due to increased snagging of polymer chains in the presence of NPs.^[^ ^39^ ^]^

**Figure 4:**
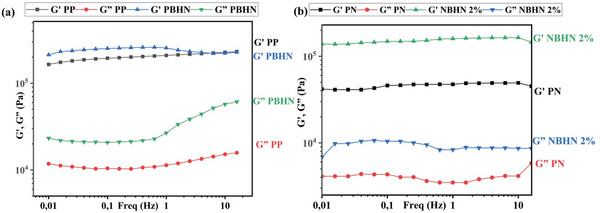
*G*′ and *G*″ of samples observed after frequency sweep from 15 to 0.015 Hz, at 1% strain and 230 °C for a) PP, PBHN, and b) PN, NBHN 2%.

The results of the frequency sweep test of the samples are illustrated in **Figures** **4**a,b. The tests were conducted at a temperature of 230 °C and a strain of 1%. The frequency was modulated between 15–0.015 Hz and the corresponding G' and G″ values were measured. For all samples the *G*′ was significantly higher than the *G*″. Throughout the analysis the *G*′ did not deviate much from the initial values. However, an increase in frequency showed a marked increase in *G*″ for PP and PBHN samples. Overall, variation in frequency at the defined parameters did not affect significantly the viscoelastic behavior of the materials, and the moduli are higher for the samples containing TiO_2_ NPs.

**Figure 5:**
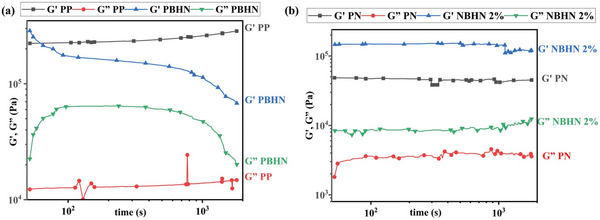
The results of time sweep analysis of a) PP and PBHN, b) PN and NBHN 2%. PP, PN, and NBHN 2% seem to retain their elastic properties, while the G' value of the PBHN falls with respect to time.

The time sweep tests of the samples reveal their behavior at constant strain over a period and are relevant for triboelectric materials as they can mimic stresses in contact‐separation mode. The resulting curves from the time sweep analysis of the samples are displayed in **Figures** **5**a,b. *G*′ of PP behaves elastically even after 1000 s, but PBHN fails to maintain *G*′ and a decrement can be seen in both loss and storage modulus. However, throughout the time sweep analysis *G*′ remained dominant for all samples. Initially, PBHN has a *G*′ value of 0.287 MPa, after 34 s it falls to the same level as PP. At the end of the test (after 1800 s) the *G*′ of PBHN reaches a minimum value of 0.07 MPa. Here, the reduction in *G*′ could be due to the alterations in relaxation times in the presence of NPs. The material might undergo a relaxation process that lowers the *G*′ values.^[^ ^40^ ^]^ Comparatively, PN and NBHN 2% show no significant change in their viscoelastic behavior and maintain the dominant *G*′ behavior within the defined parameters.

### 3.3 Fourier Transform Infrared Spectroscopy

**Figure 6:**
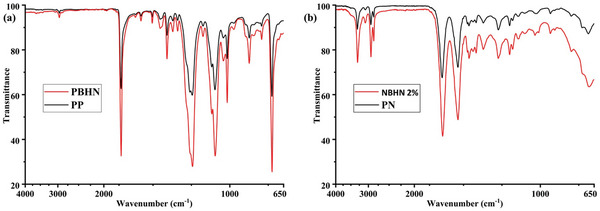
FTIR spectra for a) PP (black) and PBHN (red), b) PN (black) and NBHN 2% (red).

The FTIR spectra of PP, PN, PBHN, and NBHN 2% are presented in **Figure** **6**. In Figure 6a, the sharp peak at 1716 cm−¹ in both pristine and hybrid PET corresponds to the stretching vibrations of the C═O bond.^[^ ^41^ ^]^ Peaks at 1241 and 1093 cm−¹ are attributed to the stretching of C─O bonds in both pristine and hybrid PET.^[^ ^41^ ^]^ The small peak at 2950 cm−¹ in hybrid PET and 2935 cm−¹ in pristine PET is due to the aliphatic stretching of the C─H bond. Additionally, the sharp peak at 725 cm−¹ in both PET samples results from the out‐of‐plane bending of the C─H bond.^[^ ^41^ ^]^ A small peak at 675 cm−¹ in the hybrid PET spectrum is indicative of Ti‐O‐Ti stretching.^[^ ^42^ ^]^ In Figure 6b, the FTIR spectra of pristine and hybrid nylon 6,6 are shown. The sharp peak at 3290 cm−¹ in both samples is due to the stretching of N─H bonds.^[^ ^43^, ^44^ ^]^ The peaks at 2930 cm−¹ and 930 cm^−^¹ in pristine nylon 6,6 and the corresponding peaks at 2924 and 930 cm−¹ in hybrid nylon 6,6 are associated with the stretching of CH₂ bonds in the amide II group.^[^ ^43^, ^44^ ^]^ The peak at 1634 cm−¹ in both samples indicates the stretching of the C═O bond in the carbonyl group,^[^ ^43^, ^44^ ^]^ while the peak at 1269 cm−¹ corresponds to CH₂ stretching in the amide III group.^[^ ^43^, ^44^ ^]^ The TiO_2_ NPs have better absorption than the polymer nanofibers.^[^ ^45^, ^46^ ^]^ The sharper absorption peaks observed in the PBHN and NBHN 2% compared to PP and PN could be the result of increased absorption of infrared rays.

### 3.4 Thermal Analysis

**Figure 7:**
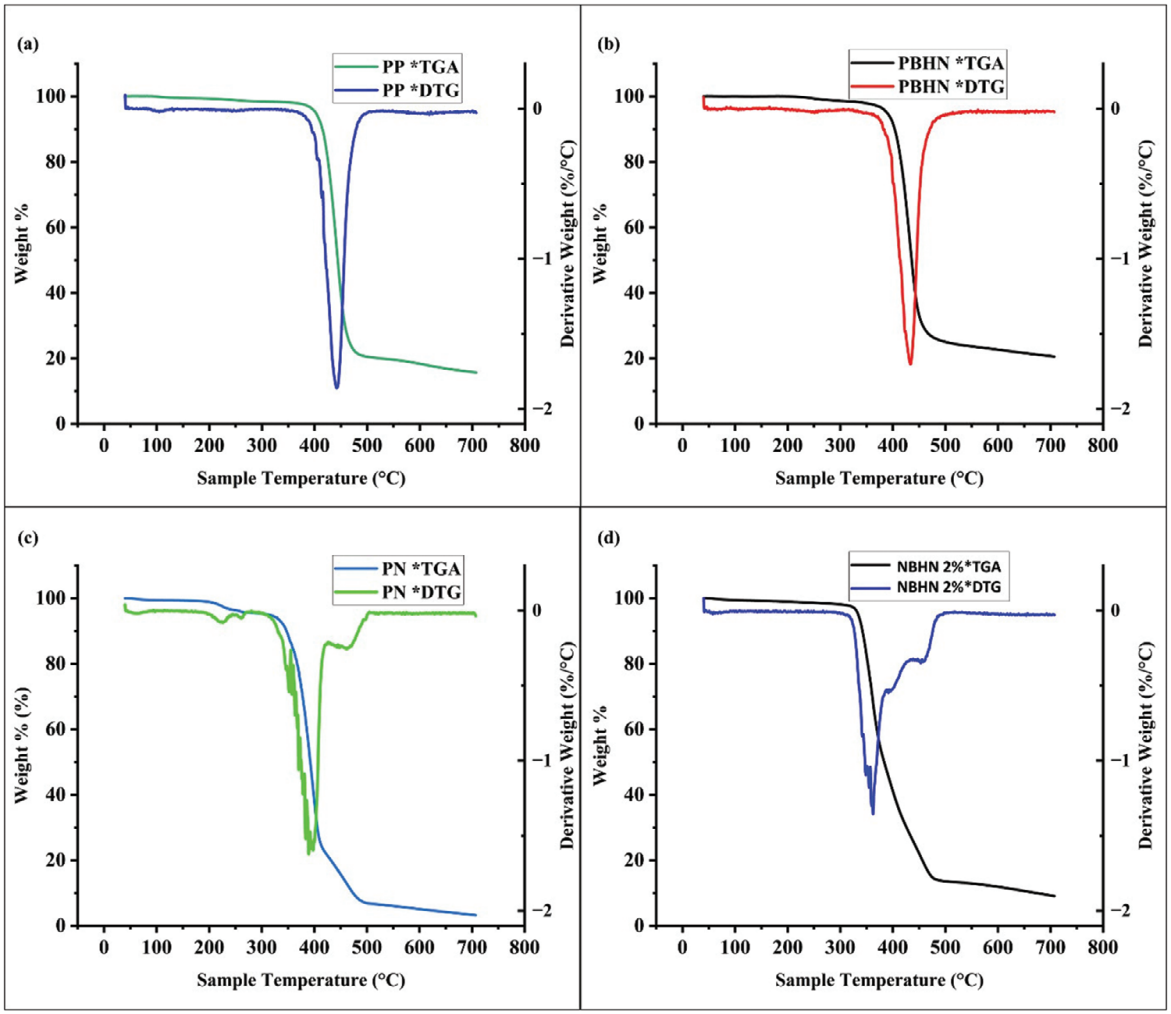
TGA and DTG curves of pristine nanofibers and nanofibers functionalized with TiO₂ NPs. a) PP, b) PBHN, c) PN, and d) NBHN 2% nanofibers.

The TGA provided significant insights into the thermal properties of both pristine and hybrid nanofibers. TENG devices harness energy wasted in the movements and often the mechanical stresses could change the temperature and lead to the performance of the TENG adversely affected. Based on the outcome of the TGA it is possible to fix the maximum working temperature for current TENG devices where the materials are still intact. The thermograms are illustrated in **Figure** **7** and the results of the TGA are shown in **Table** **2**. In the TGA curve, the weight loss of the polymer is given as a function of the temperature. The differential thermogravimetric analysis (DTG) curve is the first derivative of the TGA curve. DTG curve represents the rate of weight loss as a function of temperature and from this curve, different thermal events such as the onset of temperature and inflection point could be determined.

**Table 2:** Thermal Properties of PP, PN, PBHN, and NBHN 2% nanofibers. W_loss_, is the percentage weight loss of the polymer. T_onset_, is the temperature at which weight loss begins, and T_inflection_, is the temperature at which maximum weight loss happens.

Sample Name	W_loss_ [%]	T_onset_ [°C]	T_inflection_ [°C]
PP	85	402	442
PBHN	80	388	434
PN	97	300	390
NBHN 2%	91	334	361

The PP nanofibers experienced a weight loss of 85%, whereas the PBHN exhibited a lower weight loss of 80%. The onset point for weight loss in PP was observed at 402 °C, while in PBHN, it was observed at a slightly lower temperature at 388 °C. The inflection points were recorded at 442 °C for the PP and 434 °C for the PBHN. The weight loss showed a 2‐step process beginning at an onset temperature of 300 °C for PN and 334 °C for the NBHN 2%. The PN experienced a weight loss of 97% and the NBHN 2% experienced 91%, respectively. As the heating continued, the maximum weight loss, marked by the inflection point, occurred at 390 °C for the PN and 361 °C for the NBHN 2%, respectively.

As expected, in both PET and nylon samples, the hybrid nanofibers demonstrated less weight loss than their pristine counterparts. This reduction in weight loss is likely due to the presence of TiO₂ NPs in the hybrid nanofibers, which require a significantly higher temperature to degrade. TGA analysis of NBHN 5% showed a similar 2‐step degradation behavior as compared to the PN and NBHN 2% as seen in Figure S2 (Supporting Information). The onset temperature point for NBHN 5% was observed at 363 °C whereas the inflection temperature was 420 °C with a weight loss of 80%. As concluded from TGA curves, there is no clear trend observed for onset and inflection point due to the addition of NPs in the matrix of nanofibers. However, the total weight loss shows that the concentration of NPs in the nanofibers’ matrix is directly related to the weight of remaining materials after the end of the TGA analysis.

### 3.5 Triboelectric Performance

**Figure 8:**
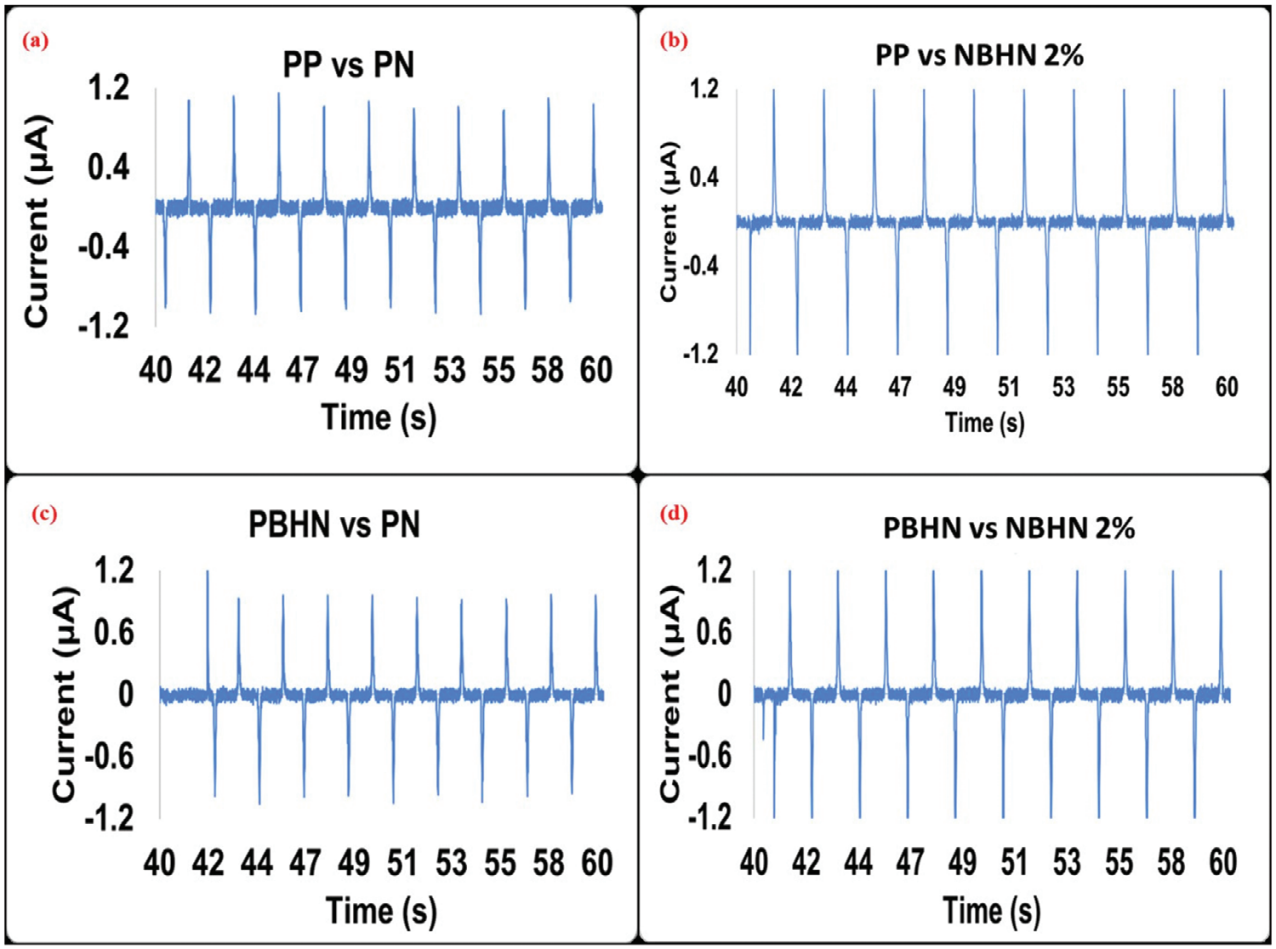
The current output of a) PP versus PN b) PBHN versus PN c) PP versus NBHN 2% d) PBHN versus NBHN 2% as contact surfaces for TENG devices at 500 mΩ impedance are shown. The current is in µA and is measured in the time interval of 40 to 60 s. The maximum output current is obtained for PBHN versus NBHN 2% with −1.643 µA.

**Figure 9:**
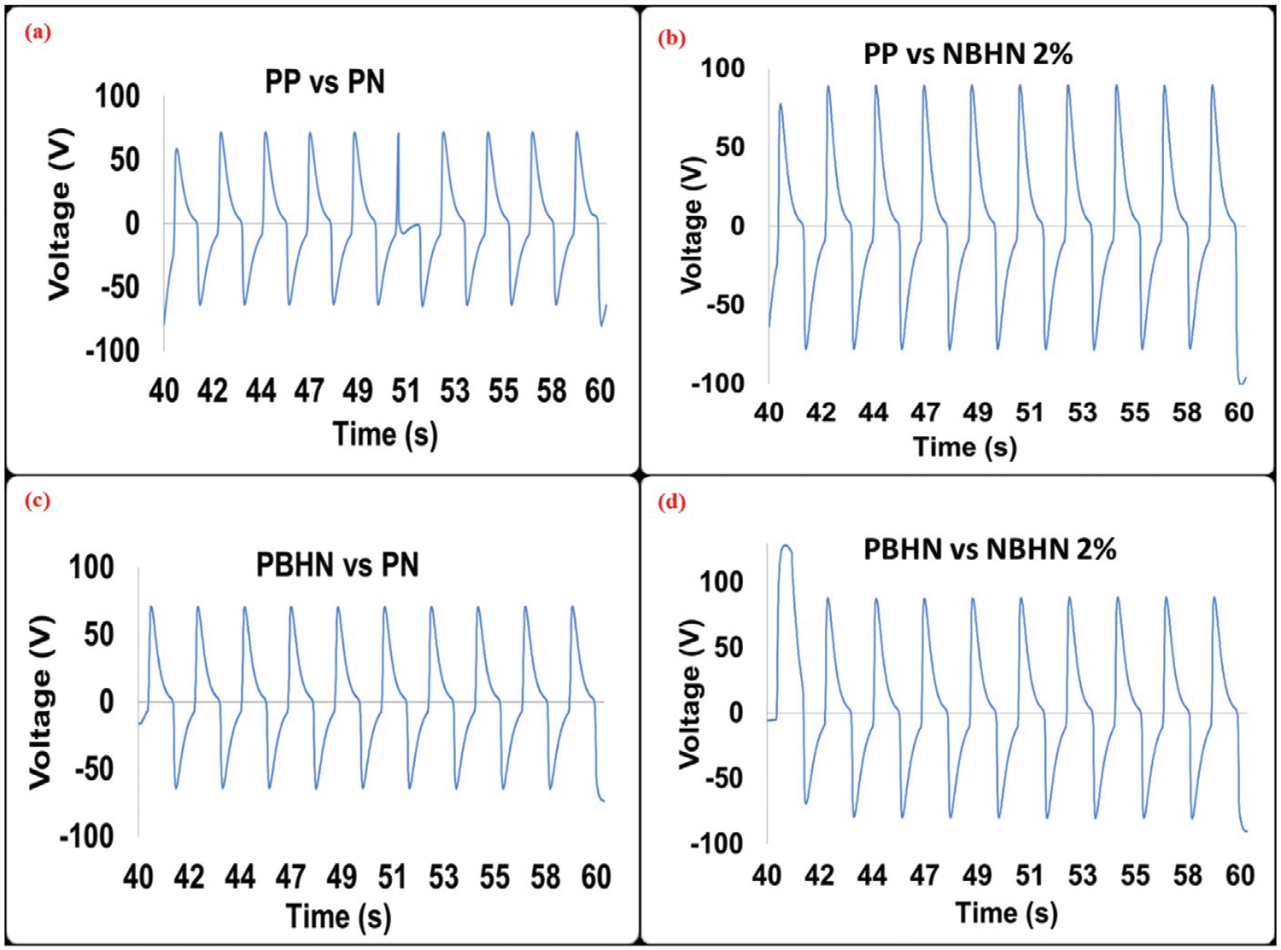
The output voltage of a) PP versus PN b) PBHN versus PN c) PP versus NBHN 2% d) PBHN versus NBHN 2% as contact surfaces for TENGs. The measurement was taken at an impedance of 500 mΩ, with PBHN/NBHN 2% TENG contact surface showing the peak voltage of −90.4 V.

**Figure 10:**
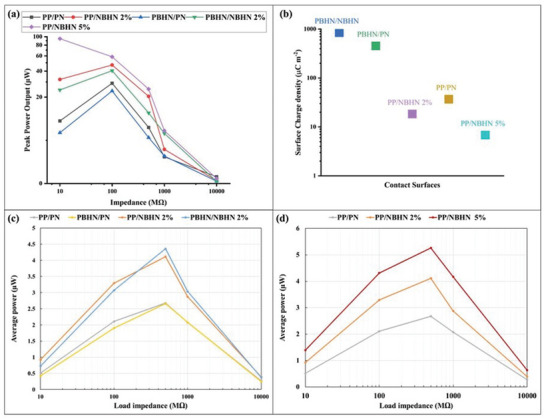
a) The peak power output of TENGs with respect to different impedance load conditions. The PP/NBHN 5% TENG has overall high output values at both 10 and 100 mΩ, followed by PBHN/NBHN 2% TENG. The highest output power is 94.92 µW for PP/NBHN 5%. b) The surface charge density of TENGs calculated are shown. The highest surface charge density is for PBHN/NBHN 2% TENG with 846 µC m^−^^2^ followed by PBHN/PN TENG with 453 µC m^−^^2^. c) The average power output of TENGs with respect to different impedance load conditions from 10 mΩ to 10 GΩ. The highest average output power is 4.359 µW for PBHN/NBHN 2% TENG, very close to 4.315 µW generated by the PB/NBHN 2% TENG. d) A comparison of the average power output of TENGs increasing the concentration of the TiO₂ NPs to 2% and 5% (w/v) of the nylon nanofibers.

To measure the triboelectric performance, various combinations of pristine and hybrid nanofibers were used at the contacting surfaces. As a result, 4 TENG devices were obtained with the combination of PP versus PN, PBHN versus PN, PP versus NBHN 2%, and PBHN versus NBHN 2% as the contact surfaces. The current and output voltage at 500 mΩ for the four TENGs are shown in **Figures** 8, 9, respectively. Some defective peaks can be observed, typically caused by momentary stiction between oppositely charged surfaces due to electrostatic forces. At this impedance, the output voltage and current achieved by the combination of PBHN/NBHN 2% TENG surfaces, with a max output voltage of −90.4 V and a max current of −1.64 µA. The combination of PP/NBHN 2% follows closely, delivering a max output voltage of −89.76 V and a max current of −1.63 µA. Surprisingly, the PP/PN TENG gives a max voltage of −80.57 V and a max current of 1.15 µA. Lastly, the hybrid PET/pristine nylon TENG records a max output voltage of −73.56 V and a max current of 1.39 µA. The power output values calculated, obtained from the measurements presented in Figures S4 and Figure S5 (Supporting Information), for all the TENGs under different load conditions, are illustrated in **Figure** **10**a, with the corresponding values provided in **Table** **3**. From Figure 10a, it is evident that both at 10 and 100 mΩ, the highest power output is achieved when a combination of PP/NBHN 5% nanofiber surfaces are used as the triboelectric materials. The PN/NBHN 2% TENG ranks second, with a slightly lower power output at both resistances, as shown in Table 3. The PBHN/NBHN 2% TENG follows in third place, then PP/PN, and the PBHN/PN TENG has the lowest power output. The peak power density could be calculated by dividing the peak power by the surface area of the TENG and for PP/NBHN 5% TENG, the value is 23.44 mW m^−2^, which is the highest among all samples.

**Table 3:** The peak power output of five TENG devices with various combinations of contact surfaces under different load impedances.

Load 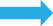 Sample Name 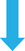	10 mΩ	100 mΩ	500 mΩ	1 GΩ	10 GΩ
P_PP/NP_ (µW)	14.48	28.94	12.98	5.74	1.28
P_PP/NBHN 2% (_µW)	49	83.1	20.41	9.15	0.78
P_PBHN/PP_ (µW)	11.75	23.87	10.82	6.07	0.64
P_PBHN/NBHN 2%_ (µW)	24.23	40.47	16.34	11.59	0.59
P_PBHN/NBHN 5%_ (µW)	94.92	58.61	24.80	12.22	1.15

The surface charge density of each TENG is depicted in Figure 10b. The highest surface charge density observed for the PBHN/NBHN 2% TENG combination reached 846 µC m^−^^2^. The PBHN/PN TENG combination follows with a value of 436 µC m^−^^2^. In contrast, the PN/NBHN 2% and PN/NBHN 5% TENG combinations exhibit significantly lower surface charge densities, at 18.38 and 6.81 µC m^−^^2^, respectively.

Overall, the improved performance of the PP/NBHN 2% and PBHN/NBHN 2% TENGs is attributed to the addition of TiO_2_ NPs in the nanofiber matrix. Specifically, when TiO_2_ NPs are blended with nylon nanofibers. As mentioned earlier, TiO_2_ NPs possess excellent dielectric properties, which, when blended with the polymer enhance the overall dielectric property of the hybrid nanofiber.^[^ ^7^, ^8^, ^9^ ^]^ This increase in dielectric property improves the triboelectric charge generation, leading to better power output.^[^ ^7^, ^8^, ^9^ ^]^ Interestingly, the effect of the addition of TiO_2_ NPs in PET nanofibers does not produce a similar effect and the average power output is almost similar to the pristine combination of nanofibers. In order to study the effect of the addition of TiO_2_ NPs to the nylon nanofibers, which clearly shows an increasing effect in the average power, we have studied an additional concentration of 5% (w/v) for the PP/NBHN combination. The results shown in Figure 10d confirm that the concentration of the TiO_2_ NPs to the nylon nanofibers is directly proportional to the generated average power which are 2.675, 4.114 and 5.265 µW for an optimal load impedance of 500 mΩ.

In this study, a significant increase in output voltage and current was observed when TiO_2_ NPs were blended with PET and nylon polymers. This finding is consistent with the results of Gulahmadov et al., who spray‐coated nylon surfaces with TiO_2_ NPs fifteen times.^[^ ^21^ ^]^ In their study, the TENG produced an output voltage of 41.38 V and a current of 2.5 µA.^[^ ^21^ ^]^ The increased current output of the nylon TENG, in contrast to the present study, could be due to the higher concentration of TiO_2_ NPs on the surface. Similarly, Park et al. embedded 5% (w/v) TiO_2‐x_ NPs in a polydimethylsiloxane‐based (PDMS) TENG.^[^ ^7^ ^]^ This TENG showed the highest output voltage and current of 180 V and 8.15 µA, respectively.^[^ ^7^ ^]^ The performance of the PDMS‐based TENG is significantly higher than that of the present study, which could be attributed to the differences in triboelectric materials and the load impedance. In 2014, Zheng et al. developed a TENG using PDMS and Kapton, achieving a peak power density of 8.44 mW m^−^^2^.^[^ ^47^ ^]^ Similarly, in 2019, Liu et al. created a 2D woven wearable TENG made of core‐shell fibers through twisting and weaving processes.^[^ ^48^ ^]^ This TENG reached a peak power density of only 2.33 mW m^−^^2^,^[^ ^48^ ^]^ which is significantly lower than the 23.44 mW m^−^^2^ achieved in the present study.

The studies by Gulahmadov et al. and Park et al. demonstrated higher output voltages and currents with higher concentrations of TiO_2_ NPs.^[^ ^7^, ^21^ ^]^ Therefore, to enhance the performance of the TENG, future work should focus on optimizing the concentration of TiO_2_ NPs further. By systematically varying the concentration and evaluating the corresponding power output, it would be possible to identify the optimal concentration that maximizes the TENG's performance while maintaining the nanofibrous structure.

Finally, to evaluate the long‐term stability of the device, performance measurements were conducted after more than 1000 operational cycles. The output voltage was measured using the maximum impedance of the source meter (10 GΩ), while the generated current was assessed at an optimal impedance of 500 mΩ to maximize power output. The results, presented in Figure S6 (Supporting Information), compare the key performance parameters before and after cycling. These data demonstrate that the device retains stable output voltage and current over extended operation, confirming its robustness and suitability for long‐term applications.

In addition to long‐term stability, the adaptability of the device to environmental conditions was assessed through output performance measurements under varying relative humidity (RH) levels. The RH was adjusted using water vapor to increase humidity and nitrogen to evacuate vapor, reducing RH levels. Measurements were performed under three conditions: a rapid RH change (1), a gradual RH change over several minutes (3), and an intermediate RH change rate (2). The results, shown in Figure S7 (Supporting Information), indicate that the output performance is highly sensitive to humidity variations. Notably, the rate of RH change influenced the output, suggesting potential hysteresis effects related to moisture absorption in the material. These findings underscore the significant impact of humidity on the device's performance and provide initial insights into its environmental adaptability. This consideration has to be taken into account when designing an operational triboelectric device, combining encapsulation strategies to keep a reduced RH around the electrodes.

## 4 Conclusion

In the present work, triboelectric materials were fabricated by electrospinning of upcycled PET and commercially available nylon. To increase the dielectric constant of the triboelectric surfaces, TiO_2_ NPs were blended with the polymer solutions to obtain hybrid nanofibers PBHN and NBHN. The average diameter of the PP and PBHN nanofibers was measured to be 274 and 565 nm, respectively. Similarly, the PN and NBHN 2% nanofibers exhibited an average diameter of 180 and 327 nm, respectively. During tensile testing, both PBHN and NBHN 2% samples displayed superior mechanical strength compared to their pristine counterparts (PP and PN). Additionally, TGA revealed that PP and PN nanofibers experienced more weight loss (≈85% and 97%, respectively) than PBHN, NBHN 2%, and NBHN 5% (80%, 91% and 80%, respectively) nanofibers samples. Finally, the TENG devices were fabricated from combinations of PP/PN, PP/NBHN 2%, PN/PBHN, and PBHN/NBHN 2% nanofiber mats. Out of these combinations, PBHN/NBHN 2% and PP/NBHN 2% showed superior performances in terms of output voltage, current, power output, and surface charge density. The peak output voltage, current, power output, and peak power density PBHN/NBHN 2% TENG in order are −90.4 V, −1.643 µA, 40.478 µW, and 16.19 mW m^−^^2^, respectively. Whereas, the peak output voltage, current, power output, and power density observed for PP/NBHN 5% TENG after an increase in TiO_2_ from 2% to 5% are 111 V, −1.61 µA, 94.92µW, and 23.44 mW m^−^^2^, respectively.

The findings of this study demonstrate the potential of using hybrid nanofibers to enhance the performance of TENGs. The use of shredded PET from upcycled bottles promotes environmental sustainability and provides a cost‐effective material for TENG fabrication. The affordability and availability of nylon and TiO_2_ NPs make this approach economically suitable for the potential large‐scale adoption. The improved mechanical strength, thermal stability, and electrical output of the TENGs indicate their advantage for various applications, including wearable electronics, energy harvesting devices, and self‐powered sensors. Alternatively, the functionalization of nanofibers with TiO_2_ NPs promises a potential platform for applications such as organic dye degradation, photocatalysis, antimicrobial membranes, and charge separator interlayer in batteries.

## Conflict of Interest

The authors declare no conflict of interest.

## Supporting information



Supporting Information

## Data Availability

The data that support the findings of this study are available from the corresponding author upon reasonable request.
