# Supplementary material for: Sustainable Electrospun Hybrid Nanofibers for Triboelectric Nanogenerators
Source: Small. 2025 Jan 29;21(9):2410271. doi: 10.1002/smll.202410271 (PMC11878251; doi:10.1002/smll.202410271)
Supplement: Supplementary file 1 — Supporting Information [file SMLL-21-2410271-s001.docx]

**Sustainable Electrospun Hybrid Nanofibers for Triboelectric Nanogenerators**

Sweetly Thomas-Kochakkadan, Marcos Duque, Gonzalo Murillo, Viraj P. Nirwan, and Amir Fahmi

**Supporting Information**


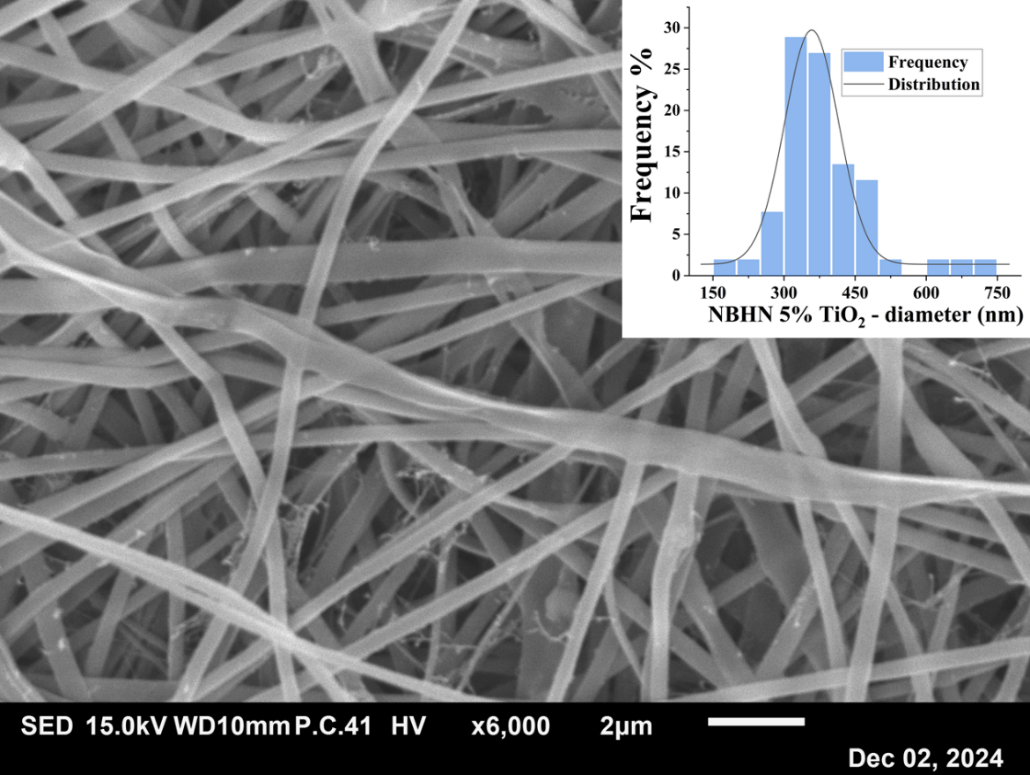


**Figure S1.** SEM micrographs of the nanofibers and the statistical analysis of the nanofiber diameter 382 ± 98 nm for nylon nanofibers functionalized with 5% (w/v) TiO₂ NPs.


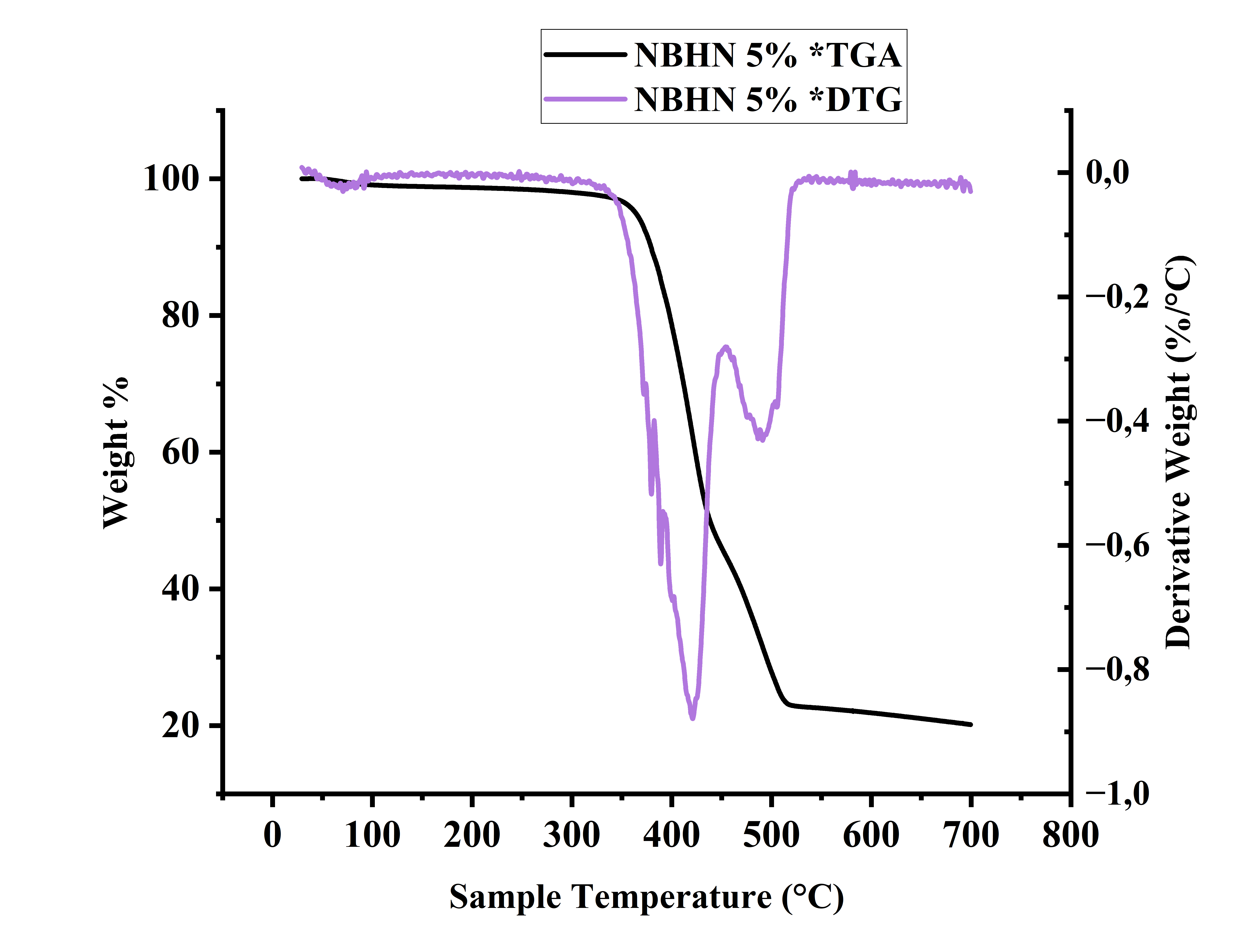


**Figure S2.** TGA and DTG curves of nylon nanofibers functionalized with 5% (w/v) TiO₂

NPs.


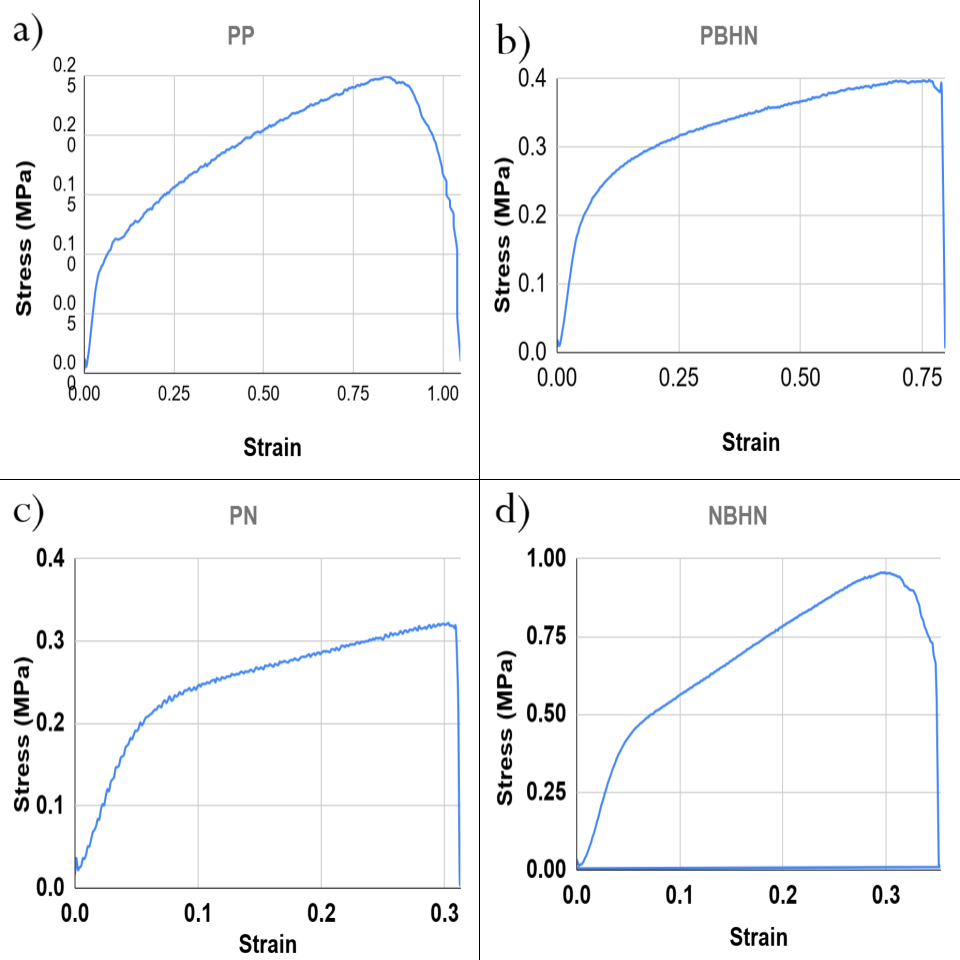


**Figure S3.** The stress-strain curves obtained from the tensile testing of a 10x20 mm strip of a) PP, b) PBHN, c) PN, and d) NBHN samples. Each curve represents the mechanical behavior of the respective polymer under tensile testing.


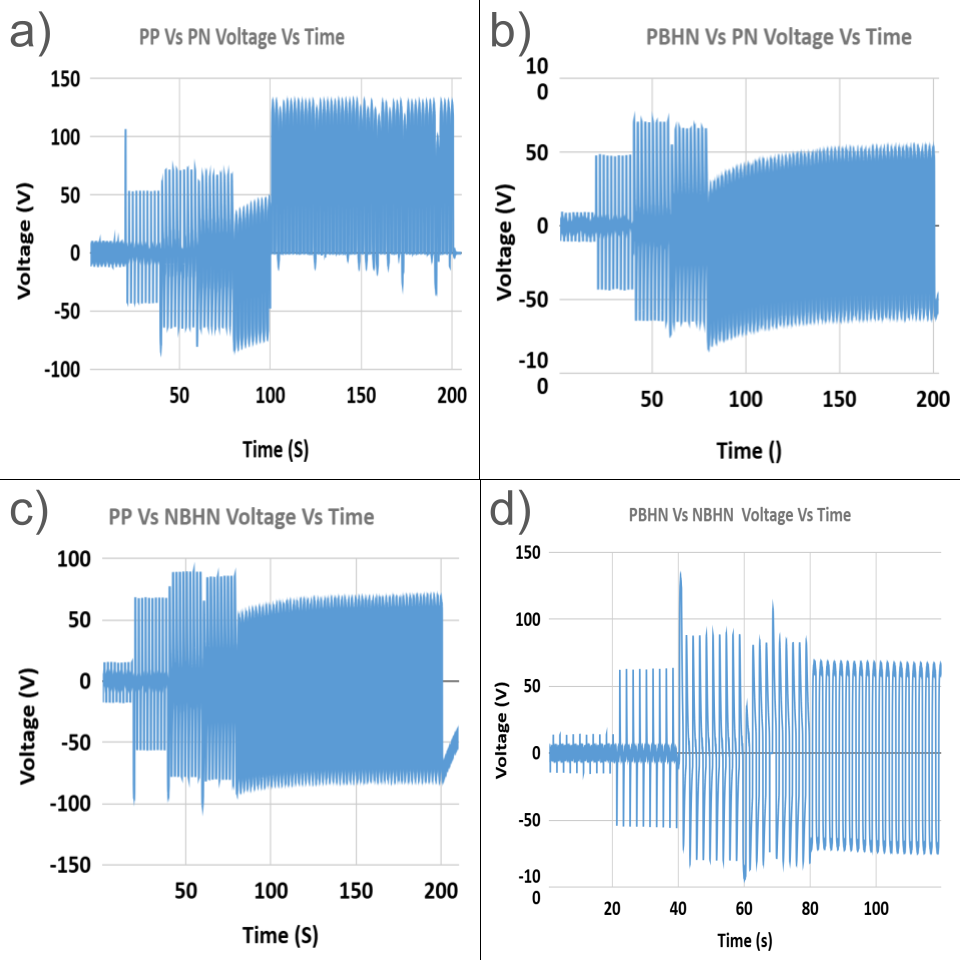


**Figure S4.** Voltage output obtained from a) PP/PN b) PBHN/PN c) PP/NBHN d) PBHN/NBHN TENG. The impedance load was changed each 20 seconds from 1 GΩ, 500 MΩ, 100 MΩ and finally to 10 MΩ.


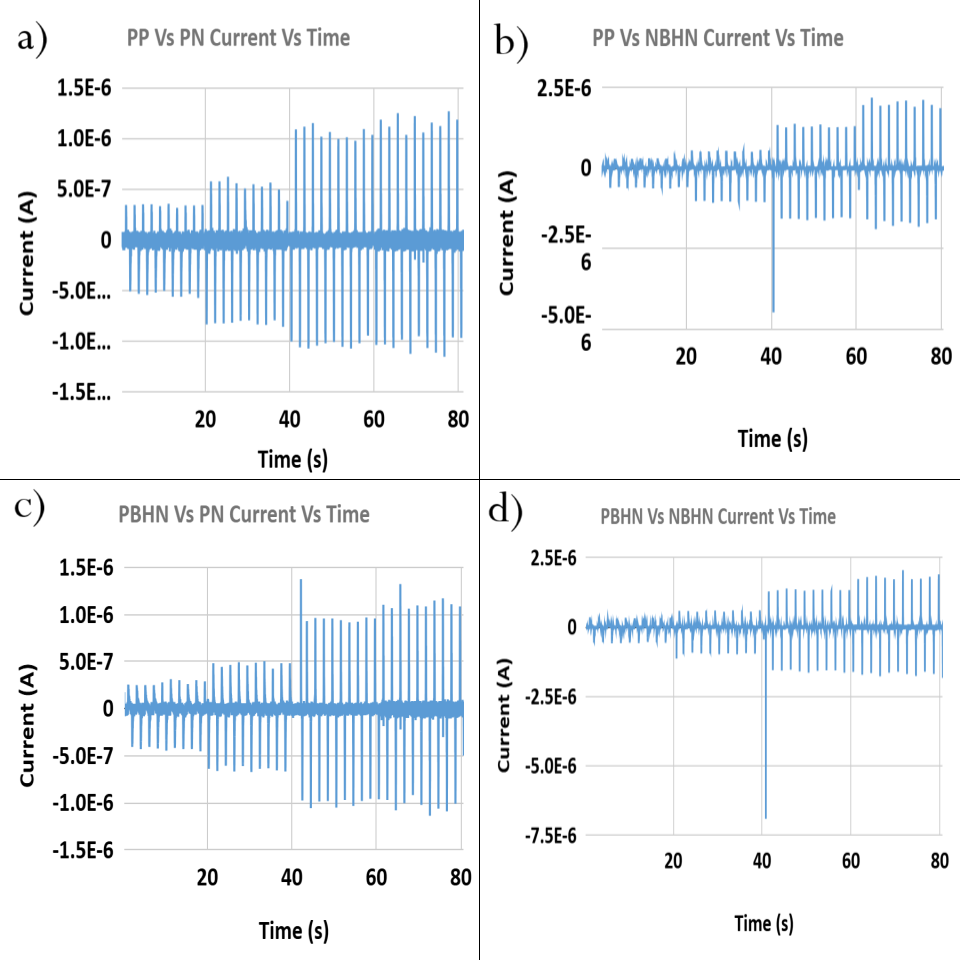


**Figure S5.** Current output obtained from a) PP/PN b) PP/NBHN c) PBHN/PN d) PBHN/NBHN TENG. The impedance load was changed in each 20 seconds from 10 MΩ, 100 MΩ, 500 MΩ, 1 GΩ and finally to 10 GΩ.


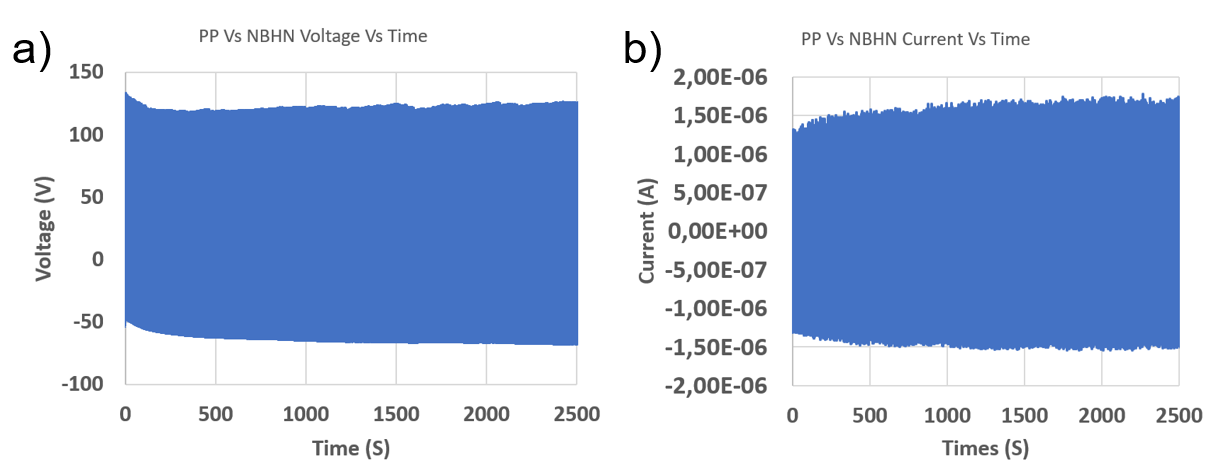


**Figure S6** a) Output voltage obtained from the PP/NBHN TENG measured with a 10 GΩ impedance over 1250 repetition cycles. b) Generated current measured with an optimal 100 MΩ impedance over 1250 repetition cycles.


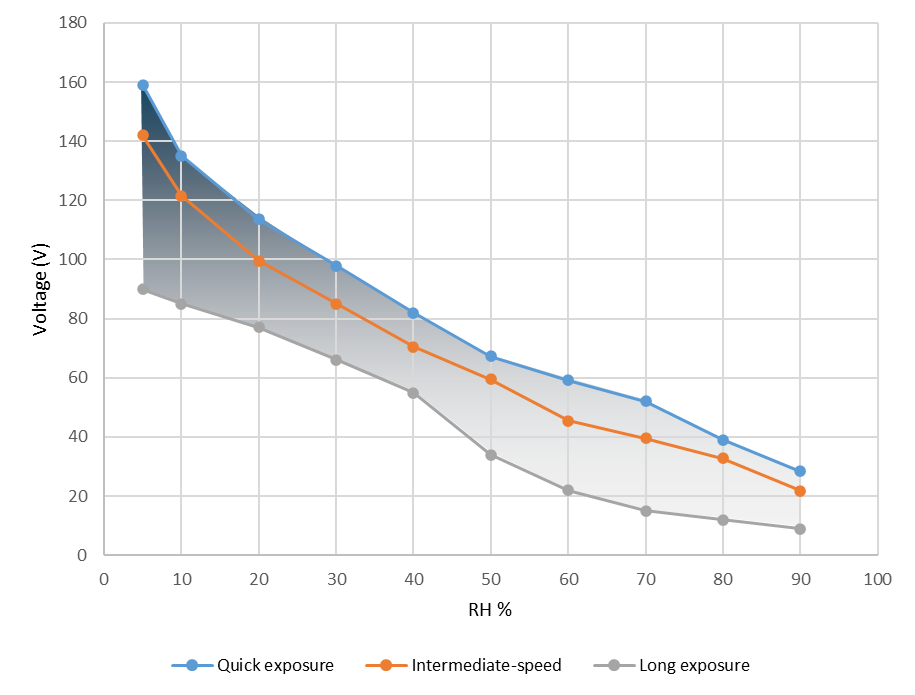


**Figure S7** Effect of relative humidity on the output voltage of electrospun fibers.
